# RNA Editing as a Therapeutic Approach for Retinal Gene Therapy Requiring Long Coding Sequences

**DOI:** 10.3390/ijms21030777

**Published:** 2020-01-25

**Authors:** Lewis E. Fry, Caroline F. Peddle, Alun R. Barnard, Michelle E. McClements, Robert E. MacLaren

**Affiliations:** 1 Nuffield Laboratory of Ophthalmology, Nuffield Department of Clinical Neurosciences & NIHR Oxford Biomedical Research Centre, University of Oxford, Oxford OX3 9DU, UK; 2 Oxford Eye Hospital, Oxford University Hospitals NHS Foundation Trust, Oxford OX3 9DU, UK

**Keywords:** inherited retinal degeneration, retinitis pigmentosa, retinal disease, RNA editing, ADAR, CRISPR, gene therapy, gene editing, base editing, genome engineering

## Abstract

RNA editing aims to treat genetic disease through altering gene expression at the transcript level. Pairing site-directed RNA-targeting mechanisms with engineered deaminase enzymes allows for the programmable correction of G>A and T>C mutations in RNA. This offers a promising therapeutic approach for a range of genetic diseases. For inherited retinal degenerations caused by point mutations in large genes not amenable to single-adeno-associated viral (AAV) gene therapy such as *USH2A* and *ABCA4*, correcting RNA offers an alternative to gene replacement. Genome editing of RNA rather than DNA may offer an improved safety profile, due to the transient and potentially reversible nature of edits made to RNA. This review considers the current site-directing RNA editing systems, and the potential to translate these to the clinic for the treatment of inherited retinal degeneration.

## 1. Introduction

**Figure 1:**
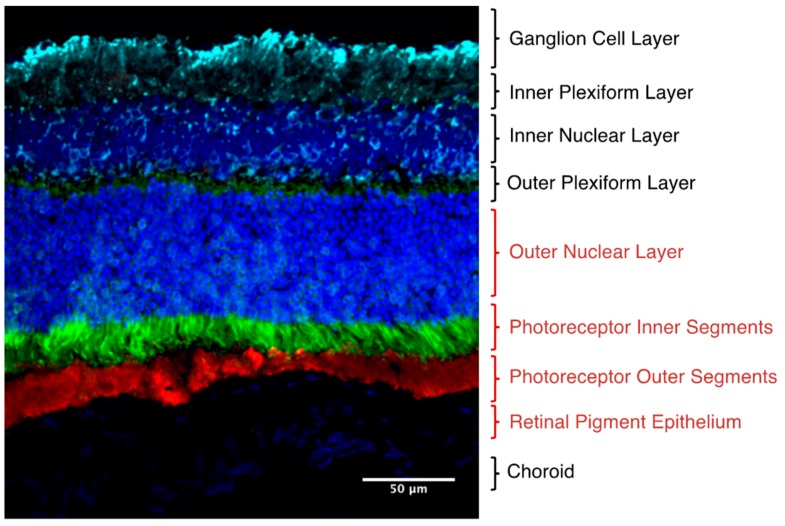
Immunofluorescence section demonstrating the anatomy of a mouse retina. Inherited retinal degenerations affect the cells of the outer retina, particularly the photoreceptors (rods and cones) and the retinal pigment epithelium, highlighted in red text. The inner (green) and outer (red) segments of the photoreceptors are demonstrated with their nuclei stained above in blue.

Programmable editing of nucleic acids offers significant therapeutic potential for a wide range of genetic diseases. The development of the Clustered Regularly Interspaced Short Palindromic Repeat–CRISPR-associated genes (CRISPR-Cas) system for facile gene editing in mammalian cells has led to a wide range of approaches for gene manipulation including gene silencing, repair and editing [1]. An area of promise for genetic therapy is for the treatment of inherited retinal degenerations. Inherited retinal diseases are an important cause of blindness that result from dysfunction and death of cells in the outer retina (Figure 1) due to mutations in a heterogenous array of genes [2]. Treatment of the underlying genetic cause of inherited retinal degenerations could halt or reverse vision loss in these patients. 

Due to the retina having relative immune privilege, easy accessibility for vector delivery and non-invasive functional and structural endpoints for assessing treatment efficacy, retinal disorders are excellent candidates for genetic therapies [3]. Gene replacement therapy to introduce the normal copy of a gene to cells lacking gene expression in X-linked and autosomal recessive conditions has shown excellent outcomes, with an FDA-approved therapy to replace the *RPE65* gene for Leber’s congenital amaurosis [4] and many others being evaluated in clinical trials [3]. Currently, adeno-associated viral (AAV) vectors are the most commonly used delivery mechanism to express genes in retinal cells [3]. AAV vectors offer high tropism for photoreceptors and RPE, the commonly targeted cells in retinal degeneration [5]. Furthermore, their low immunogenicity and the safety profile established across multiple clinical trials make them attractive delivery vehicles.

For the many patients who have a disease-causing mutation in genes too large for the ~4.7 kb packaging capacity of AAV, however, a different treatment strategy is required. The cDNA for genes such as *ABCA4* and *USH2A* do not fit in a single AAV, and in the USA mutations in these two genes account for 25% of families with inherited retinal degeneration [6]. While alternative approaches using other delivery strategies such as using dual AAV vectors, alternative viral vectors or non-viral vectors are being investigated [7,8], a substantial number of inherited retinal degenerations still cannot be treated with AAV gene therapy. Furthermore, titrating transgene expression in the retina can be challenging, and overexpression of transgenes from strong promoters may lead to cellular toxicity, making it desirable to edit the endogenous gene [9,10,11,12].

Gene editing aims to correct the endogenous genetic sequence rather than delivering a replacement gene. The DNA editing capabilities of enzymes of the class II CRISPR systems such as Cas9 and Cas12 have been widely used for programmable RNA-guided DNA targeting [1]. In the eye, this has been used to knockdown dominant genes in animal models [13,14,15,16], reprogram photoreceptors [17], target angiogenic factors [18] and excise a deep intronic splicing mutation in the *CEP290* gene, a strategy moving forward to clinical trial this year [19]. Correction of coding mutations in large genes using DNA editing requires a different approach. Strategies that require the introduction of a correct donor template, such as homology-directed repair of double-stranded breaks have low in vivo efficiency [20,21,22]. Approaches such as DNA base editing [23] or prime editing [24] can relatively efficiently introduce specific corrections without double-stranded breaks but use large constructs that are not currently deliverable with AAV vectors. While efforts to improve in vivo editing efficiencies and reduce off-target mutations continue, the threat of introducing permanent off-target mutations in DNA elsewhere throughout the genome is a significant challenge for all DNA targeting strategies [25].

Unlike DNA, messenger RNA (mRNA) molecules exist transiently within the cell and transmit the genetic information that encodes for the production of proteins. A strategy to edit RNA rather than DNA is, therefore, highly appealing, as this allows for the editing of pathogenic mutations at a transcript level, without the risk of creating permanent off-target mutations in the genome. Furthermore, the transient nature of RNA means that RNA editing is potentially reversible and controlled over time.

Recent advances in RNA editing technologies have enabled the development of engineered enzymes capable of either adenosine-to-inosine (A-I) or cytosine-to-uracil (C-U) edits. Site-directed RNA editing uses molecular tools to recruit RNA editing enzymes to target sites of interest and enables the targeted correction of G>A and T>C mutations in coding sequences of RNA. Given that G>A and T>C mutations comprise up to 61% of all pathogenic point mutations in humans [26], these tools are an exciting therapeutic prospect for inherited retinal degeneration.

Here, we review the potential for using RNA editing enzymes for site-directed RNA editing, and how this might be applied to correct mutations in large genes implicated in inherited retinal degenerations not amenable to AAV gene therapy.

## 2. ADARs: Adenosine Deaminase Acting on RNA

### 2.1. ADAR Expression, Structure and Function

**Figure 2:**
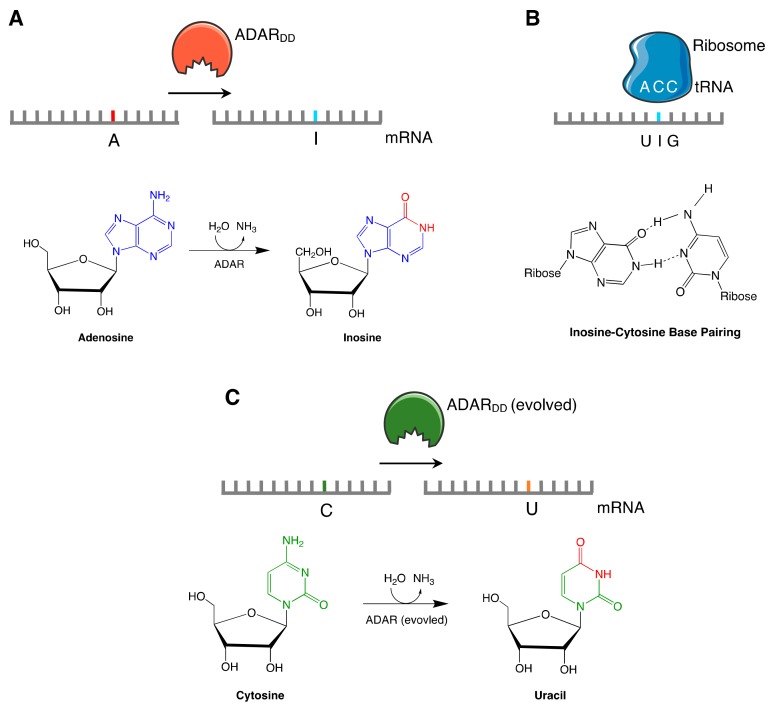
(**A**). Demonstrates the hydrolytic deamination of adenosine to inosine. (**B**) Base pairing between inosine and cytosine allow inosine to be read as a guanosine during translation by the ribosome, effectively an A-G edit. (**C**). Evolved mutants of human ADAR allow for deamination of cytosine to uracil for C-U editing. ADAR = adenosine deaminase acting on RNA; DD = deaminase domain.

The majority of strategies to edit RNA utilize the activity of the naturally occurring human adenosine deaminase acting on RNA (ADAR) enzymes. ADARs act to convert adenosine residues to inosines (A>I editing) in double-stranded RNA. Inosine is structurally similar to guanosine and is read as a guanosine by most cellular machinery including during translation, splicing and reverse transcription, effectively creating an A>G edit in RNA [27] (Figure 2). 

Two ADAR enzymes have been identified in humans to carry out A>I editing activity, ADAR1 and ADAR2. ADAR1 is expressed as two isoforms, a shorter 110kDa isoform (ADAR1 p110) and a larger 150 kDa isoform (ADAR1 p150). ADAR1 p110 is constitutively expressed and localizes to the nucleus, while ADAR1 p150 is inducible by activation of the innate immune sensing system and localizes to the cytoplasm [28]. ADAR1 is expressed ubiquitously including in the retina [27,29]. ADAR2 is expressed predominantly as a single isoform and nuclear localization signals mediate the localization of ADAR2 primarily to the nucleus and nucleolus [30]. ADAR2 is most strongly expressed in the lung and brain [27,29]. Evidence suggests that ADAR2 RNA is expressed in the retina and immunohistochemistry demonstrates ADAR2 localised to retinal ganglion cells. However, protein expression in other cells types has not been demonstrated [29,31]. Given that editing of pre-mRNA is observed and can be dependent on double-stranded RNA structures formed by introns, it is likely that RNA editing of mRNA occurs in the nucleus, before or concurrently with splicing [27,32]. 

A>I editing one of the most common post-transcriptional modifications in humans [27]. Most A>I editing occurs in non-coding sequences, including in the 5′ and 3′ untranslated regions (UTRs) and within intronic retrotransposon units such as Alu and long-interspersed elements (LINE). In mammals, many of these regions are repetitive regions and editing of repetitive regions is principally mediated by ADAR1 [33]. A>I editing in coding sequences occurs more rarely, but can lead to codon changes and amino acid alterations [27]. Editing in these non-repetitive regions is principally mediated by ADAR2 [33]. This site-specific editing of coding sequences is strikingly demonstrated in the glutamate receptor *Gria2* (also known as GluR2) in mice [34]. Loss of ADAR2 recoding of the Q/R site is lethal post-natally, and rescue results in viable animals [34]. Altered expression of ADAR or changes to tissue A>I editing activity has been associated with a range of diseases [35]. However, the physiological role of the majority of ADAR editing beyond *Gria2* remains to be elucidated [36].

All ADARs have two common structural motifs. A double-stranded RNA-binding domain (dsRBD) makes direct contact with double stranded RNA (dsRNA), while a C-terminal deaminase domain (ADAR_DD_) catalyses hydrolytic deamination where an amino group is replaced by a hydroxyl group, converting adenosine to inosine (Figure 2). This allows inosine to act as guanosine and pair with cytidine by Watson-Crick base-pairing. A base-flipping mechanism rotates the target adenine base from the RNA helix into the enzyme catalytic pocket to allow the enzyme to attack the target C6 carbon [37]. 

In natural perfectly matched duplex RNA, ADARs deaminate approximately 50% of adenosines. However, factors such as the local sequence context and complex secondary structures can affect editing rates [38]. Wildtype ADARs have a preference for adenosines with a nearest neighbour 5′-A or -U and a 3′-G as interactions of these near-neighbour nucleotides with the deaminase domain appear to promote stabilization of the flipped base [37].

### 2.2. Engineering ADARs for RNA Editing

#### 2.2.1. A-I Editors

To improve A-I editing efficiency for genome engineering, rational and random mutagenesis has produced a number of ADAR mutants. The E488 residue of ADAR2 stabilizes the base-flipped structure by taking the space of flipped adenosine and hydrogen bonding to the opposite base [37]. The E488Q hyperactive mutant displays increased editing activity, likely because glutamic acid (Q) is relatively more protonated than glutamine (E) and thus makes it a better hydrogen bond donor to bind an opposing cytidine base [37]. Common to most strategies, it was identified that ADAR editing preferentially occurs of adenosines that are mismatched with a cytidine as the opposite base in the guide RNA target (rather than a thymidine), and this A-C mismatch improves editing efficiency at the target site [39]. Conversely, an A-G mismatch reduces editing efficiency [40]. In the ADAR1_DD_, the E1008Q mutation has been identified as a hyperactive mutation with similar activity to the E488Q mutation in ADAR2_DD_ [41,42].

Further engineering has produced variants with improved specificity. The T375 residue helps stabilize the edited strand in the cleft containing the active site through hydrogen bonding to the 2’-hydroxyl of the flipped base and the phosphate between the flipped base and the +1 base [37]. An engineered E488Q/T375G ADAR2_DD_ mutant displays much greater specificity and relatively preserved efficiency for editing of the target adenine [43]. 

#### 2.2.2. C-U Editors

To develop the scope of RNA editing to other bases, Abudayyeh et al. developed a deaminase capable of cytosine (C) to uracil (U) RNA editing by undertaking mutagenesis of the ADAR2_DD_ [44]. Using insights from the structural homology of human ADAR2 and the naturally occurring cytosine deaminase from *Escherichia Coli*, they undertook rational mutagenesis of the hyperactive human ADAR2_DD_, and then subsequently evolved this using random mutagenesis to yield a C to U editing ADAR_DD_(C-U), which retains A-I editing capabilities. 

Further mutagenesis was used create a more specific ADAR_DD_(C-U) mutant with a S375A mutation to yield a higher specificity variant with fewer A-I off-targets. This S375A substitution is at the same amino acid position as the higher specificity ADAR2_DD_ mutant (T375G), supporting the role of this position in ADAR_DD_ A-I editing specificity [44]. Unlike A-I ADAR editing used in a number of systems, these C-U editors have only been utilized so far in the RESCUE CRISPR-Cas13 based system described later. 

## 3. RNA Editing with ADARs 

First proposed by Woolf and colleagues almost 25 years ago [45], the A-I editing activity of ADARs can be used for site-directed RNA editing. Recently, many new approaches have been developed to harness these enzymes. In order to develop a treatment for an inherited retinal degeneration caused by mutations in a long gene, it is necessary to understand and evaluating the many site-directed RNA editing strategies and mechanisms across a broad range of disease applications tested to date. 

**Figure 3:**
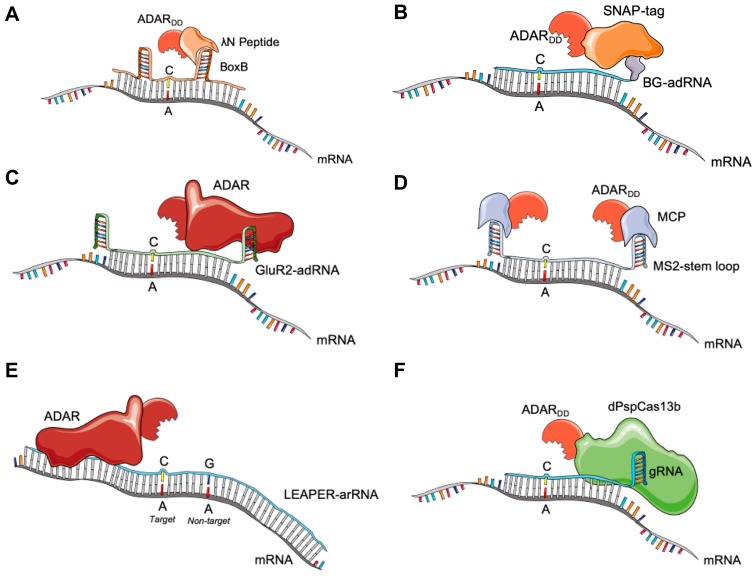
Schematic figures demonstrating the RNA editing approaches of (**A**) BoxB-λN-ADAR with a dual BoxB design to recruit the λN peptide (**B**) SNAP-tag-ADAR fusion with a O6-benzyl-guanine (BG) conjugated adRNA (**C**) Glu-adRNA approach where the GluR2 R/G hairpin recruits exogenous or endogenous full length ADAR (**D**) MS2-MCP-ADAR stem-loop approach with dual MS2 stem-loop hairpins recognized by the MS2 bacteriophage coat binding protein (MCP) (**E**) An example of endogenous ADAR recruitment using long LEAPER-arRNAs and G-A mismatches within the guide region to reduce off-target editing (**F**) The REPAIR system with dPspCas13b-ADAR fusions recruited by a guide RNA with a direct repeat. Note the A-C mismatch at the target adenosine in each strategy to promote editing at the target site. gRNA = guide RNA; adRNA = ADAR guiding RNAs; λN = Lambda N protein; LEAPER = Leveraging Endogenous ADAR for Programmable Editing of RNA; REPAIR = RNA Editing for Programmable A to I Replacement; dPspCas13b = deactivated Cas13b orthologue derived from *Prevotella sp. P5-125*.

To enable site-directed ADAR editing, it is necessary to create a double-stranded RNA structure at the target region and draw the ADAR to this site. Strategies, therefore, broadly comprise two parts: (1) a guide RNA (gRNA) sequence antisense that can hybridize to the target RNA, and (2) an effector mechanism that recognizes the gRNA and recruits the ADAR enzyme. These strategies may recruit the whole native ADAR enzyme including its dsRNA binding domains or express only the deaminase domain (ADAR_DD_). Strategies using the whole ADAR enzyme may utilize the naturally expressed endogenous ADAR or introduce this with exogenous expression. Strategies using the ADAR_DD_ typically fuse this to another protein effector that is capable of programmable RNA binding to enable site-directed RNA editing. Figure 3 graphically illustrates each approach, while Table 1 present a summary of the results obtained with each tool discussed. 

### 3.1. BoxB-λN-ADAR

One of the first demonstrations of engineering ADAR for site-directed RNA editing used the interaction between the BoxB RNA hairpin and the Lambda N protein (λN). First described in bacteriophage as an anti-terminator of transcription [46], the λN protein naturally binds short stem-loop RNA structures called BoxBs. For site-directed RNA editing, the 22 amino acid binding site of the λN protein was fused to the ADAR2_DD_ (Figure 3A). A guide RNA incorporates BoxB hairpins in the middle of an antisense guide sequence with a mismatched C at the target adenosine. Binding of the λN peptides to the BoxB-gRNAs recruits the ADAR_DD_ to the editing sites. Montiel Gonzales et al. reported that in *Xenopus* oocytes injected with the cDNA of the cystic fibrosis gene *CFTR* containing a premature stop codon, the λN-BoxB-ADAR2_DD_ system had a 20% correction efficiency with partial functional restoration of the CFTR protein [47]. Subsequent optimization of this system has improved editing rates, including by using four copies of the λN binding peptide and using two rather than one BoxB hairpin in the gRNA structure to achieve 20–70% efficiency editing reporter constructs in HEK293T cells [48]. 

Sinnamon et al. have also employed a similar BoxB-λN system delivered via AAV to edit the *Mecp2* gene involved in Rett syndrome in primary murine neurons [49]. Using an AAV1/2 vector containing a single λN binding peptide fused to an ADAR2_DD_ (E488Q) together with a BoxB-gRNA expression cassette containing six copies of the guide, they demonstrated a 72% on-target correction of a missense *Mecp2* mutation and partial restoration of MECP2 protein [49]. 

A significant drawback of λN-BoxB systems has been substantial off-target editing events, especially with the 4λN system with ADAR2_DD_ (E488Q) [50]. Use of a nuclear localization signal (NLS) to target primary RNA transcripts in the nucleus and lower amounts of guide RNA may reduce these [50]. 

### 3.2. SNAP-ADAR

The SNAP-ADAR system uses a SNAP-tag fused to an ADAR_DD_ [51]. SNAP-tag enzymes are engineered to recognize O6-benzyl-guanine (BG) as a substrate and form a covalent linkage. Stafforst and colleagues generated modified nuclease-resistant guide RNAs conjugated to BG [52]. The BG-guide RNA is covalently bound by the SNAP-ADAR_DD_ fusion via the SNAP-BG reaction, and this guides the deaminase to the RNA target (Figure 3B). A recent study has demonstrated that with hyperactive mutants of the ADAR1 and two deaminase domains, impressive editing efficiencies of up to 90% of endogenous transcripts have been observed in a HEK293 cell line expressing SNAP-ADAR under a doxycycline-inducible promoter [53]. Editing rates were significantly influenced in this study by near-neighbour preferences, with target bases with 5′G demonstrating much lower rates of editing. A significant drawback of this system, however, is that due to the chemical modifications required for the guide RNA, it is not genetically encodable for stable viral delivery, for example within AAV. Clinical translation would likely require repeated administration of the guide RNA as used in antisense oligonucleotide therapeutics. If penetration of the gRNA to the relevant cell (e.g., photoreceptors) that express a SNAP-ADAR construct is feasible, this would theoretically allow for adjustable dosing much like a traditional drug. 

### 3.3. GluR2-ADAR

The GluR2-ADAR system uses a naturally occurring GluR2 sequence to recruit the full-length ADAR2 protein. The GluR2 mRNA hairpin (termed an R/G motif) is a naturally occurring substrate strongly recognized by the dsRBD of ADAR2. This was harnessed to engineer an adRNA (ADAR-associated RNA, functionally equivalent to a gRNA) composed of the R/G motif fused to an antisense guide sequence. The dsRBD of a full-length ADAR protein recognizes the R/G motif and is recruited to the target site (Figure 3C). First demonstrated by the Stafforst group with plasmid delivery of wildtype ADAR2, editing rates of up to 10% in the Parkinson’s associated gene *PINK1* were reported in HeLA cells [54]. Recent further work by Katrekar et al. used ADAR2(E488Q) overexpression together with a targeting adRNA, enabled on-target editing rates of endogenous transcripts of 10–40% in HEK293T cells [42]. 

As the full length ADAR2 protein is only 2.1 kb, it can be packaged in AAV together with the GluR2-adRNA sequences. Katrekar et al. attempted targeted correction of G>A mutations in two mouse models using AAV8-ADAR2-GluR2adRNA constructs [42]. The correction of a stop codon in the *mdx* mouse model of Duchenne’s muscular dystrophy required the editing of two adenosines to correct a termination codon (TAA) to tryptophan (TGG). Intramuscular AAV injection of GluR2-adRNA + ADAR2 (both with and without the E488Q mutation) resulted in TAA>TGG correction in ~0.8% of RNA transcripts and dystrophin partial protein restoration. In the second model, the authors targeted a G>A splice variant in the *spf^ash^* mouse model of ornithine transcarbamylase (OTC) deficiency. Systemic injection of GluR2-adRNA + ADAR2 resulted in 4.6–8.2% correction of the pre-mRNA and a reduction in the incorrectly spliced fraction of mRNA, confirmed by a 2.5–5% restoration of ornithine transcarbamylase protein. Correction rates were noticeably higher in those systemically injected with the E488Q mutant. However, the authors observed significant toxicity in these mice not observed in those injected with the wildtype ADAR. While the mechanism for this toxicity has not been established, it raises concerns for the systemic use of the E488Q mutant. 

### 3.4. MS2-MCP-ADAR

Recruitment of the ADAR deaminase domain has also been achieved using the bacteriophage-derived MS2-MCP tagging system. The MS2 bacteriophage coat protein (MCP) naturally binds the MS2 stem loop from its genome. This MS2 stem loop can be attached to a short guide sequence to create an MS2-adRNA, and this adRNA recruits an MCP-ADAR_DD_ fusion protein to the editing site of interest (Figure 3D). Katrekar et al. evaluated a MCP-MS2 system using an adRNA composed of a 20 nucleotide guide sequence with a mismatched C in the 6th position flanked by an MS2 stem-loop structure each side of the guide [42]. Systematic evaluation of the on-target efficiency using Sanger sequencing and RNA-seq analysis of different MCP-ADAR_DD_ constructs on eight endogenous transcripts in HEK293T cells found that both MCP-ADAR1_DD_ and MCP-ADAR2_DD_ constructs could achieve editing rates that ranged from 10% to 80% in both coding and untranslated regions. Constructs with an ADAR1_DD_, a nuclear export signal (NES), and deaminases with hyperactive mutations each demonstrated higher editing efficiencies. However, each of these factors also contributed to higher off-target editing rates. In an *mdx* muscular dystrophy mouse model, an intramuscularly delivered AAV8-MCP-ADAR1_DD_(E1008Q)-NLS construct delivered with an MS2 gRNA demonstrated 2% on-target efficiency and partial restoration of dystrophin expression [42]. 

Another group found that an MCP-ADAR1_DD_ system that used a 21 nucleotide gRNA with a single MS2 stem loop was found to have a 7% editing efficiency for a premature termination codon in an EGFP reporter system in HEK293 cells [55]. This paper did not investigate the use of features known to optimize RNA editing efficiency such as the use of a mismatched C in the gRNA at the target nucleotide, the use of nuclear localization or export signals, or linker sequences for the MCP-ADAR_DD_ fusion. 

### 3.5. Endogenous ADAR Approaches 

Although editing rates are noticeably improved by the overexpression of exogenous ADARs, evidence from a number of recent papers suggests that recruitment of endogenous ADAR to edit double-stranded RNA is a potentially viable strategy. 

Initial work by Fukuda and colleagues demonstrated that A to I editing of RNA molecules could be achieved using ADAR guiding RNAs (AD-RNA) and recombinant ADAR in an in vitro reaction [56]. The custom AD-RNA was constructed with a short stem-loop structure and further developed with insights from the design of the GluR2 hairpin. They demonstrated that this system could be used in HEK293 cells to edit GFP expression constructs and repair a premature stop codon [56]. 

The LEAPER editing system (Leveraging Endogenous ADAR for Programmable Editing of RNA) developed by Qu et al. utilizes their observation that expression of long RNA (71–191 nucleotide) guides creates regions of dsRNA long enough to enable endogenous ADAR1 binding and editing [40]. LEAPER guides were designed without any domains for ADAR recruitment, and use a single A-C mismatch at the target site to direct editing (Figure 3E). Guanosine mismatches placed at non-target adenosine sites reduce off-target editing within the long guide binding region. Plasmid expression of a 151 nucleotide guide from a U6 promoter was used to target a range of endogenous targets in HEK293 cells, with editing rates of up to 50% in the 5′UTR and reduced editing rates in coding regions of up to 20%. In primary cells lines editing rates of 30–80% were achieved in the 5′UTR of *PPIB*, a chosen candidate endogenously expressed gene that encodes a cyclophilin enzyme. Long 111 nucleotide guides, chemically stabilized with 2′-O-methyl and phosphorothioate linkage modifications, were also electroporated into fibroblasts derived from patients with Hurler syndrome (a severe form of mucopolysaccharidosis type 1) with a Trp402Ter mutation. This restored previously deficient IDUA enzyme activity and was found to have higher A>I editing activity and enzyme activity restoration when targeting pre-mRNA (30%) compared to mRNA (10%). Lentiviral delivery of LEAPER guides was also attempted but only achieved 6% editing rates in the 5′UTR of *PPIB* in HEK293 cells [40]. 

In contrast to the long guides of the LEAPER system, the RESTORE editing system (Recruiting Endogenous ADAR to Specific Transcripts for Oligonucleotide-mediated RNA Editing) recently published by Merkle and co-workers uses shorter 20–40 nucleotide chemically modified anti-sense oligonucleotides (ASO) with an R/G ADAR recruiting domain to edit transcripts with endogenous ADAR1 [57]. Using 20 nucleotide ASOs as guides, editing rates of the 3′UTR of *GAPDH* of 4–34% across a range of immortalized cell lines and 10–63% in primary cell lines were observed, but editing in the open reading frame was not possible. Further engineering and lengthening (40 nucleotide) of the ASO enabled a 10–20% correction of an α-1-antrypsin deficiency mutation in the open reading frame of the *SERPINA1* gene in HeLa cells, and a 7–21% editing rate of tyrosine 701 in the *STAT1* gene in primary fibroblasts and RPE. They noted all editing rates could be improved by induction of expression of the ADAR1p150 isoform by administration of interferon alpha [57]. 

From these studies, it appears that the recruitment of endogenous ADAR is possible either with long gRNAs or with recruitment domains. Minimal editing activity is seen when using antisense guides without recruitment domains that are less than 60 nucleotide [42] or 70 nucleotide [40]. As with other RNA based strategies, delivery of a stable RNA that can be genetically encoded may be challenging. Further work to optimize these parameters and evaluate them with an appropriate delivery strategy in vivo is required. In vivo recruitment of endogenous ADAR has been observed, although the correction rates were almost undetectable (~0.6%) in OTC mice injected with a short 20 nucleotide AAV-GluR2-adRNA alone without ADAR overexpression [42]. 

### 3.6. CRISPR-Cas13 for RNA Editing 

#### 3.6.1. A to I Editing 

The type VI CRISPR nuclease Cas13 binds to single stranded RNA directed by a programmable CRISPR RNA (crRNA) guide [58,59,60,61]. In their active form, the Cas13 enzyme family (Cas13a-d) [62] act as ribonucleases (RNase) following recognition of a target RNA sequence [58,59,60,63,64,65,66,67,68], cleaving both target RNA transcripts and surrounding bystander transcripts with a generalized RNase activity in bacteria. In human cell lines, this collateral cleavage effect has not been observed [43,63] but the underlying mechanism for this observation remains to be determined. RNase activity is mediated by two higher eukaryotes and prokaryotes nucleotide-binding (HEPN) RNase domains. Mutation to key conserved catalytic residues in the two HEPN domains deactivates the RNase activity of Cas13, resulting in a catalytically deactivated protein capable of programmable RNA binding (dCas13) [43,58,59,68].

Using the capacity of dCas13 to create targeted double-stranded RNA substrates, Cox and colleagues used a Cas13b orthologue derived from *Prevotella sp. P5-125* (PspCas13b) fused to ADAR2_DD_ to create a programmable RNA editor termed REPAIR (RNA Editing for Programmable A to I Replacement) [43]. A guide RNA comprised of a spacer sequence and a 3′ direct repeat region is used to direct dCas13b binding to the sequence of interest (Figure 3F). Guides with homology regions of between 30 and 84 nucleotides and with an A-C mismatch to the target adenosine placed across a range of positions within the homology region were found to be functional. However, 50 nucleotide guides with a mismatch placed 32–36 nucleotides from the 5′ end was found to be generally most efficacious. 

A key advantage of Cas13 is that, unlike Cas9 and Cas12 nucleases, Cas13 does not require a protospacer adjacent motif (PAM) sequence local to the gRNA binding site [59,63]. Although evolved Cas9 variants have now created a wide range of PAM specificities [69], this remains an important restriction in the targeting range of Cas9, particularly for base editing. In bacterial screens, Cas13a and Cas13b enzymes exhibit a variety of preferences for nucleotide motifs 5′ and 3′ to the homology region, termed protospacer flanking sequences (PFS) [70]. For Cas13b RNA knockdown and editing in human cell lines, however, a preferred PFS sequence could not be identified from experiments using a library of PFS sequences [43]. The lack of requirement for a PFS greatly broadens the targeting scope of Cas13b mediated base editing. 

REPAIRv1 uses the hyperactive ADAR2_DD_(E488Q) (dPspCas13b-ADAR2_DD_(E488Q)) and displayed editing rates of 10–40% across a range of overexpressed gene fragments and up to 29% of two endogenously expressed RNA transcripts [43]. The REPAIRv1 construct also appears to have a broad codon scope, with relaxed near-neighbour flanking nucleotide preferences compared to those observed with wildtype ADAR. Whilst the nucleotides 5′ and 3′ to the target A do influence the editing rate, editing rates between 10% and 30% were observed for all combinations of flanking sequences. 

As previously observed, the hyperactive ADAR2_DD_(E488Q) can produce numerous off-target editing events, so further rational mutagenesis was undertaken to generate a more specific ADAR2_DD_(E488Q/T375G) deaminase fused with dPspCas13b (termed REPAIRv2). This modification produced a 900-fold decrease in off-target events, however, with an approximate 2-fold loss of on-target efficiency [43]. This double mutant specific ADAR_DD_ has not been evaluated in many other systems but was reported to have low efficiency in the SNAP-tag system [53].

The direct repeat hairpin of the gRNA is proposed to recruit the Cas13-ADAR fusion to the target site. Vogel and colleagues found that they could achieve low editing rates with a 50 nucleotide guide without the 35 nucleotide direct repeat (DR) region using the REPAIRv1 construct [53]. RNA editing rates are markedly improved by inclusion of the DR, however [53], and in further work, Abudayyeh et al. did not observe A-I editing using the RESCUE system [44] (discussed below) in 30 or 50 nucleotide guides without the direct repeat. In another study 70 nucleotide Cas13-gRNAs were sufficient to allow endogenous ADAR editing in the dsRNA region formed by the guide [42]. The mechanism to explain these conflicting findings remains unresolved and may occur due to the hyperactivity of the ADAR_DD_-E488Q mutant editing at dsRNA sites. It is likely, however, that a majority of the RNA editing rates observed using Cas13-ADAR editing are due to recruitment by the guide RNA. 

RNA editing with REPAIR constructs has been directly compared to other genetically encodable RNA editing systems relevant for clinical use. REPAIRv2 (dCas13b-ADAR_DD_-E488Q/T375G) was compared to the λN-BoxB-ADAR_DD_ (E488Q) and similar on-target efficiencies were observed but as expected when using the E488Q ADAR, λN-BoxB had substantially more off-targets [43]. Direct comparison of on-target activity with similar GluR2 and Cas13b constructs with hyperactive ADAR2 against the same endogenous targets revealed similar editing efficiencies [42].

#### 3.6.2. C to U Editing

RNA Editing for Specific C to U Exchange (RESCUE) is a cytosine (C) to uracil (U) RNA editing platform based on the work of Abudayyeh et al., created through serial mutagenesis of the ADAR_DD_ to create the ADAR_DD_(C-U) described earlier. Fused with a different Cas13 orthologue, deactivated Cas13 from *Riemerella anatipestifer* (dRanCas13b), the resultant dRanCas13b-ADAR2_DD_(C-U) was named “RESCUE”. They reported C-U editing efficiencies of up to 30% of endogenous transcripts and up to 42% of synthetic reporter constructs in HEK293FT cells. RESCUE editing was also used to activate STAT and Wnt/β-catenin pathways and alter cell proliferation phenotypes through C-U editing of key phosphorylation sites in transcripts in HEK293FT and human umbilical vein endothelial cells (HUVECs). The RESCUE editor still maintains A-I editing activity and is capable of multiplexed editing of the same transcript with different guides to achieve both A-I and C-U editing. As seen with REPAIR, RESCUE also demonstrates A-I off-targeting, as well as C-U off-targeting although to a lesser extent. 

Further mutagenesis was used to create a S375A mutation in the ADAR_DD_(C-U), and this system was termed RESCUE-S. This yielded an approximately 12-fold reduction in A-I off-targets and ~1.8-fold reduction in C-U off-targets while maintaining specificity. 

#### 3.6.3. Synthetic CRISPR-Like RNA Editors 

Recent work has also been undertaken to engineer a synthetic RNA targeting system made from human-derived proteins that mimic the gRNA binding, ssRNA recognition and effector functions of Cas13 proteins [71]. The CRISPR-Cas-inspired RNA targeting system (CIRTS) fuses effector proteins including ADAR2, to a gRNA hairpin binding protein and a protein designed for ssRNA binding. The CIRTS8 system using ADAR2_DD_(E488Q) was delivered via plasmid transfection to HEK293T cells and demonstrated 47% efficiency repairing a premature termination codon in a luciferase reporter. While more work is required to validate this in other cells lines, on endogenous targets and in vivo, due to its small size (<3kb), the CIRTS system could easily be delivered with AAV [71]. 

## 4. RNA Editing for Large Inherited Retinal Genes

### 4.1. Gene Targets for RNA Editing in Inherited Retinal Degeneration

In theory, base editing might be used to correct any gene implicated in inherited retinal degeneration with G>A or T>C single-base mutations. Some diseases may be more amenable to RNA editing than others, however. Dominant diseases require knockdown or correction of mutant alleles. It is likely that for dominant diseases, in vivo editing rates higher than those that are currently observed will be required for a therapeutic effect, while small recessive genes are probably more effectively treated with AAV gene replacement. The most immediate application, therefore, is for recessive genes larger than ~4.2 kb, which are unlikely to be treatable with AAV-mediated gene replacement. 

The most common large genes implicated in recessive inherited retinal degeneration and their relative frequencies in the cohort of 1000 consecutive patients diagnosed with inherited retinal degeneration by Stone et al. are shown in Table 2 [6]. Patients with mutations in genes with sizes larger than the coding capacity of AAV make up 28% of the patients within the cohort, with *ABCA4* and *USH2A* mutations accounting for the vast majority. 

### 4.2. Distribution of Targetable Mutations with RNA Editing 

**Figure 4:**
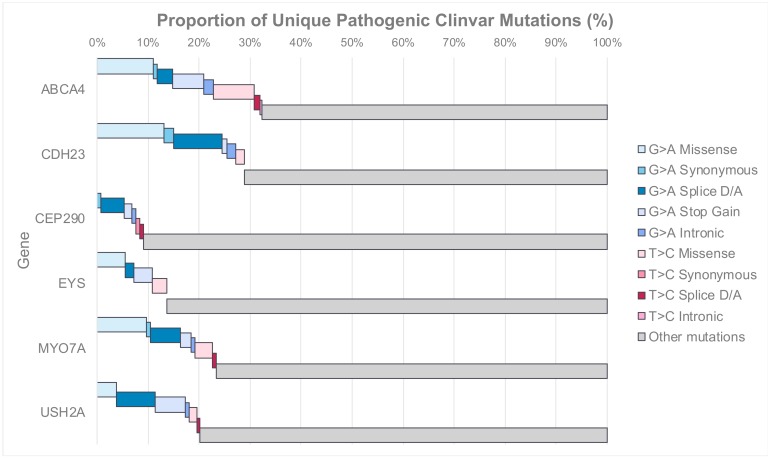
The distribution of unique “editable” mutations within all variants classed as “pathogenic” or “likely pathogenic” within the ClinVar Database. This includes all mutations including insertions, deletions and copy number variation, and these are included in “other mutations”. Blue bars represent G>A variants, while pink bars represent T>C variants. Accessed 3 October 2019.

Of all mutations in these genes, a proportion are amenable to single-nucleotide base editing with the currently available tools. G>A mutations are among the most common single-nucleotide mutations, with over 20,000 in the genome [43]. An evolutionary bias towards transition mutations, where bases remain as purines (G,A) or pyrimidines (C,T), over transversions that change bases from purines and pyrimidines may explain why these mutations are more prevalent [72]. To investigate the landscape of mutations across large retinal genes, we analysed all unique variants classed as “Pathogenic” or “Likely Pathogenic” in the ClinVar database (Figure 4) [73]. The proportion of targetable mutations (either G>A or T>C) range from 9% in *CEP290* to 32% in *ABCA4*. Many of the mutations within *ABCA4* are missense mutations (18%), while donor/acceptor splice mutations (8%) and the creation of premature stop codons (6%) are more common unique pathogenic mutations in *USH2A*. Overall, there are a greater proportion of G>A than T>C mutations. In both *ABCA4* and *USH2A*, there are at least 75 unique pathogenic G>A mutations. 

Of all targetable mutations, G>A mutations in tryptophan codons that produce premature stop codons such as TGG>TGA/TAG/TAA (p.Trp>Ter) are the most obvious initial targets as they are more easily defined as pathogenic, and restoration of the full-length mRNA should restore protein expression. In theory, the spliceosome will recognize inosine at the same strength and manner as guanosine if donor/acceptor splice sites are repaired, but this remains to be further investigated. Missense mutations will need to be carefully targeted on a case by case basis. 

### 4.3. A Case Study of RNA Editing for Inherited Retinal Disease

**Figure 5:**
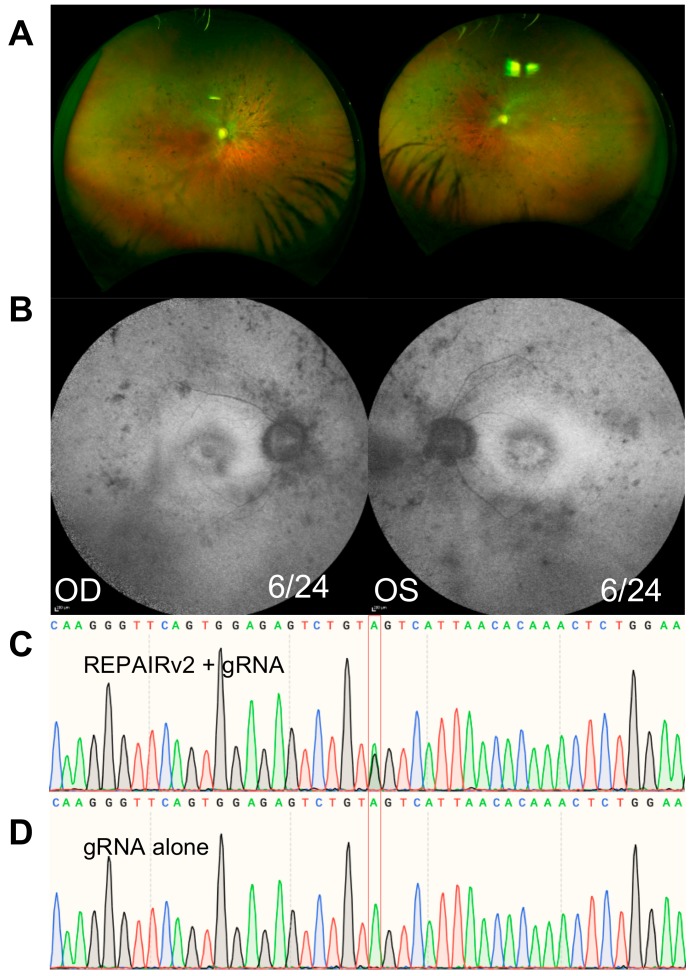
An approach to RNA editing in a patient with Usher Syndrome type 2 with compound heterozygous mutations in *USH2A* (c.2299delG / c.11864G>A). (**A**) Fundus photographs and (**B**) fundus autofluorescence images demonstrating pigmentary retinopathy and narrowing of the retinal vasculature. (**C**) Following expression of an *USH2A* cDNA fragment in HEK293T cells, the 11864G>A (p.Trp3855Ter) mutation can be partially repaired with an efficiency of 43% to restore the premature termination codon to tryptophan. The target adenosine is highlighted in grey. (**D**) Repair of the target is not observed without expression of the REPAIRv2 (dPspCas13b-hDAR2_DD_(E488Q/T375G)) plasmid.

A 50-year-old female presented for further investigation of her deteriorating vision. Diagnosed with congenital bilateral moderate deafness aged four, she subsequently developed increasing nyctalopia and was diagnosed with syndromic retinitis pigmentosa at age 15. Her parents and children are unaffected, but her brother experienced deafness and vision loss from an early age. On assessment, she was bilaterally pseudophakic, and her best corrected visual acuity was reduced to 6/24 bilaterally. Fundus examination revealed moderate pigmentary retinopathy and narrowing of the retinal vasculature (Figure 5) and optical coherence tomography showed outer retinal thinning. She had grossly constricted visual fields and a non-recordable ERG. Sequencing revealed heterozygous mutations in *USH2A*: the common c.2299delG mutation and the c.11864G>A which result in a codon change from tryptophan (TGG) to a premature termination codon (TAG). 

Given that this was a targetable mutation with RNA editing, we cloned a synthetic sequence of exon 60 of *USH2A* cDNA encoding the c.11864G>A mutation into a plasmid driven by a ubiquitous promoter. A 50 bp guide RNA with a 32 bp distance between the direct repeat and a mismatched cytosine was designed to target the mutation. HEK293T cells were transfected with the *USH2A* plasmid, guide RNA and the REPAIRv2 expression construct dPspCas13b-hDAR2_DD_(E488Q/T375G). At 48 h post transfection, cells were harvested, and isolated RNA was sent for Sanger sequencing. Analysis of the Sanger sequencing peak heights with trace decomposition revealed 43% correction of the target adenosine, repairing the termination codon to tryptophan. No off-target editing of adjacent adenosines was observed 100 bp either side of the mutation. 

As RNA editing rates are incomplete, ranging from 1% to 80% editing across different targets, cell lines and expression systems, diseases where partial restoration of a functional protein are required are ideal targets. While the level of correction required for the adequate expression of Usherin protein expression remains unknown, given the large size and stability of the Usherin protein in the photoreceptor cilium, it is possible that only a small amount of protein is required to improve or slow down degeneration.

### 4.4. Towards Clinical use of RNA Editing 

#### 4.4.1. Clinical Considerations of RNA Editing

The potential for RNA editing has now been demonstrated in vitro and in vivo for pathogenic mutations in genes related to cystic fibrosis, Duchenne’s muscular dystrophy, Hurler’s syndrome, and Ornithine transcarbamylase (OTC) deficiency, among others [42,47,49,54,74]. 

RNA editing may be advantageous compared to genome editing when moving towards clinical translation. Due to the transient nature of RNA transcripts, RNA editing is potentially reversible or titratable depending on the formulation or delivery of the editing system. For example, inducible RNA editors, systems with a built in “off” switch, or systems that require continual dosing such as using ASOs, have inbuilt safety mechanisms that allow a finer degree of control than DNA editing. This is balanced, however, against the attractiveness of a single-treatment DNA editing approach, and different approaches may suit different clinical scenarios. 

As ADAR proteins are of human origin, there are less concerns for their immunogenicity, although this may still be a factor with construct containing elements such as the viral origin MS2-MCP and bacterial origin CRISPR-Cas systems. Immune reactions to editing proteins may neutralize their editing efficiency or cause potential harmful immune responses. 

For therapeutic efficacy and safety, sufficient and persistent on-target editing will need to be balanced against rates of off-target editing. Although off-target effect will occur in transitory transcripts, further work is required to understand where and how often these off-targets are likely to occur and what phenotypic effects these may have. While off-targeting editing often occurs in non-coding regions or causes a silent variant that results in a synonymous amino acid, editing of off-target sites may cause undesired changes in the transcriptome.

#### 4.4.2. Endogenous and Exogenous ADAR Strategies

Using endogenous ADAR strategies are attractive as this limits concerns for immunogenicity and appear to have lower off-target editing events compared to strategies using exogenous ADAR [40,57]. It remains unknown how efficacious an endogenous strategy may be in vivo, however. For clinical applications in the retina, further work to understand the expression and activity of endogenous ADAR across cell types is required. While ADAR editing has been observed in the inner retina [31,75], little is known about photoreceptors or RPE. Further, formation of dsRNA regions is similar to RNA interference mechanisms, where a small dsRNA is used to induced targeted RNA degradation [76]. While in the LEAPER system RNA interference was not observed [40], this was observed by Katrekar et al. using long 100 bp adRNAs [42]. 

For the correction of pathogenic mutations, editing of the coding sequence is required. As natural ADAR editing predominantly occurs in the UTR, it is not surprising that strategies using endogenous ADAR editing demonstrate higher efficiency in the UTRs and editing is limited in the coding sequence. Editing in the coding sequence may be reduced by translation, as inhibition of translation with puromycin has been noted to increase A-I editing [53]. Furthermore, editing scope will likely be limited by the codon preferences of endogenous ADARs, and endogenous ADAR strategies cannot mediate edits other than A-I. 

Constructs expressing and recruiting exogenous ADAR or its deaminase domain with shorter gRNAs offer a number of advantages compared to endogenous strategies. Exogenously delivered ADAR may overcome reduced targeting scope of endogenous ADAR due to the codon preferences and potentially are better able to edit the open reading frame. Furthermore, exogenous strategies may benefit from future enzyme engineering work to expand the base targeting possibilities from solely A-I editing, as demonstrated by the recently developed C-U editors. The robust on-target editing rates observed with all of these exogenous systems are counterbalanced by relatively high off-target rates both within the guide region and throughout the transcriptome. Ongoing optimization will likely reduce this, as seen with the development of the E488Q/T375G variant. Protein localization may also play an important role in the balance between on-target and off-target editing. Natural ADAR editing likely occurs in the nucleus or nucleolus. In experiments investigating active Cas13 knockdown of RNA without a fused ADAR construct, higher RNA cleavage rates are observed when Cas13b is fused to a NES rather than a NLS [43], and a similar effect is seen with other ADAR-based RNA editing tools [42,50]. An advantage of nuclear localized ADAR editors is that these may reduce the frequency of off-target editing of flanking adenosines [42,50]. Evidence from other Cas13 constructs suggests that Cas13 may localize poorly to the nucleus using common NLS signals such as a single SV40 NLS and the nucleoplasmin NLS, and other more effective NLS strategies may need to be explored [77]. 

#### 4.4.3. Delivery Challenges

Delivery of both DNA and RNA base-editing constructs continues to be a challenge for therapeutic use. AAV-mediated delivery is the most readily clinically translatable delivery route, and low rates of editing using AAV-MCP-MS2 and GluR2 systems have been achieved in muscle [42]. The MCP and GluR2 systems are small, and a key advantage is that they are easily packaged in a single AAV capsid. An AAV-Cas13b-ADAR_DD_ construct packaged with appropriate tissue specific promoters targeting natively expressed transcripts has yet to be demonstrated. Cas13b orthologues identified to date for RNA targeting in mammalian cells are already relatively small (~3.2–3.5 kb) and appear to tolerate C-terminal truncations with little loss in RNA editing activity. Fused with the ADAR_DD_ (1.15 kb), CRISPR-RNA editors delivered via a single AAV are a viable option, unlike the large DNA base editor constructs. Although the full-length REPAIR fusion (dPspCas13b-ADAR2_DD_) is 4.47 kb and unable to fit in the AAV coding capacity together with the required regulatory elements, the dCas13bΔ984-1090-ADAR2_DD_ variant (4.15 kb) has relatively preserved editing efficiency compared to the full length construct and is theoretically small enough to package in AAV with short regulatory sequences. The C-terminal truncated mutant tested with the RESCUE system, dRanCas13b-del892-1095 is the smallest identified Cas13b-ADAR used for RNA editing to date (3.88 kb) and maintained similar editing efficiency to the full-length protein. Efforts to identify smaller Cas13 orthologues are ongoing. For methods requiring chemical stabilization of the RNA guide [40,53], AAV delivery of the guide is not possible, but there is potential for this to be delivered separately to the ADAR construct.

## 5. Conclusions

RNA editing is an exciting therapeutic prospect for the treatment of point mutations in genes implicated in inherited retinal degeneration that are too large for gene replacement therapy with AAV. The growing armament of tools for RNA editing will allow the interrogation of a range of approaches for safety and efficacy in vivo. As this requires a mutation-specific approach, it will be important to elucidate design principles for guide RNAs in order to expand this to a wide range of individual mutations in their individual sequence context. 

## Figures and Tables

**Table 1:** Comparison of each RNA editing tools and their on-target efficiencies for endogenous transcripts. Editing efficiencies of reporter systems or over-expressed are not reported here. ADAR = adenosine deaminase acting on RNA; λN = Lambda N protein; MCP = MS2 bacteriophage coat binding protein; REPAIR = RNA Editing for Programmable A to I Replacement; RESCUE RNA Editing for Specific C to U Exchange; LEAPER = Leveraging Endogenous ADAR for Programmable Editing of RNA; RESTORE: Recruiting Endogenous ADAR to Specific Transcripts for Oligonucleotide-mediated RNA Editing; AAV = adeno-associated virus.

System		Immortalized Cells	Primary Cells	In Vivo	Ref
**Exogenous ADAR**					
BoxB-λN-ADAR	Gene	*Mecp2*	*Mecp2*	Not tested	[47,49,50]
	Model	N2A	Murine neuron		
	Efficiency	25–50%	72%		
	Delivery	Plasmid	AAV		
SNAP-ADAR	Gene	Many targets	Not tested	Not tested	[53]
	Model	HEK293			
	Efficiency	Up to 90%			
	Delivery	Guide transfection			
MS2-MCP-ADAR	Gene	Many targets	Not tested	*Dmd*	[42,55]
	Model	HEK293T		*mdx* mouse	
	Efficiency	10–80%		AAV	
	Delivery	Plasmid transfection		2%	
GluR2-ADAR	Gene	*PINK1*		*Dmd, Otc*	[42,54]
	Model	HELA HEK293T	Not tested	*mdx* mouse model *spf^ash^* mouse model	
	Efficiency	10–40%		0.8% (*mdx*) 4.6–8.2% (*spf^ash^*)	
	Delivery	Plasmid transfection		AAV	
REPAIR: CRISPR-Cas13b-ADAR (A-I)	Gene	*KRAS, PPIB*	Not tested	Not tested	[43]
	Model	HEK293FT			
	Efficiency	28%			
	Delivery	Plasmid			
RESCUE: CRISPR-Cas13b-ADAR (C-T)	Gene	Multiple	*CTNNB1*	Not tested	[44]
	Model	HEK293FT	HUVEC cells		
	Efficiency	3–42% in 5% when multiplexed	Not reported		
	Delivery	Plasmid transfection	Plasmid transfection		
**Endogenous ADAR**					
LEAPER (long gRNA)	Gene	Many targets	Many targets	Not tested	[40]
	Model	Multiple lines	Multiple lines		
	Efficiency	Up to 50% (plasmid) 6% (lentivirus)	30–80%		
	Delivery	Plasmid Lentivirus	Plasmid electroporation		
RESTORE (ASO, chemical modification)	Gene	Many targets	*GAPDH, STAT1, SERPINA1*	Not tested	[57]
	Model	Many cell types	Multiple lines		
	Efficiency	3–34%	Up to 27%		
	Delivery	ASO Transfection	ASO Transfection		
GluR2	Gene	Not tested	Not tested	*Otc*	[42]
	Model			*spf^ash^* mouse model	
	Efficiency			0.6%	
	Delivery			AAV	

**Table 2:** Six most commonly implicated genes in recessive inherited retinal degeneration with coding sequences greater than the packaging capacity for a single AAV. ^#^ Analysis of data from Stone et al. [6] on cases in the USA.

Gene	Frequency (%) ^#^	Coding Sequence Length (kb)
*ABCA4*	17.3	6.81
*USH2A*	7.6	15.6
*CEP290*	1.8	7.44
*MYO7A*	0.8	6.65
*EYS*	0.6	9.43
*CDH23*	0.4	10.1
Total	28.5	

## Abbreviations

AAV	Adeno-associated virus
ADAR	Adenosine deaminase acting on RNA
ADAR_DD_	Deaminase domain of ADAR
*BoxB-λN*	BoxB RNA hairpin-Lambda N protein
CRISPR	Clustered Regularly Interspaced Short Palindromic Repeat
Cas	CRISPR-associated genes
CIRTS	CRISPR-Cas-inspired RNA targeting system
dsRBD	Double-stranded RNA-binding domain
HEK	Human embryonic kidney
HEPN	Higher eukaryotes and prokaryotes nucleotide-binding domain
HUVEC	Human umbilical vein endothelial cells
LEAPER	Leveraging Endogenous ADAR for Programmable Editing of RNA
MS2-MCP	MS2 bacteriophage coat protein
NLS	Nuclear localization signal
NES	Nuclear export signal
PAM	Protospacer adjacent motif
PFS	Protospacer flanking sequences
REPAIR	RNA Editing for Programmable A to I Replacement
RESCUE	RNA Editing for Specific C to U Exchange
RESTORE	Recruiting Endogenous ADAR to Specific Transcripts for Oligonucleotide-mediated RNA Editing
RNA	Ribonucleic acid
UTR	Untranslated region
